# Methylome-dependent transformation of *emm*1 group A streptococci

**DOI:** 10.1128/mbio.00798-23

**Published:** 2023-07-10

**Authors:** Joana Alves, Joshua D. Rand, Alix B. E. Johnston, Connor Bowen, Nicola N. Lynskey

**Affiliations:** 1 The Roslin Institute, University of Edinburgh, Easter Bush Campus, Midlothian, Scotland, United Kingdom; University of Arizona, Tucson, Arizona, USA

**Keywords:** bacterial transformation, restriction modification system, group A *Streptococcus*

## Abstract

Genetic intractability presents a fundamental barrier to the manipulation of bacteria, hindering advancements in microbiological research. Group A *Streptococcus* (GAS), a lethal human pathogen currently associated with an unprecedented surge of infections worldwide, exhibits poor genetic tractability attributed to the activity of a conserved type 1 restriction modification system (RMS). RMS detect and cleave specific target sequences in foreign DNA that are protected in host DNA by sequence-specific methylation. Overcoming this “restriction barrier” thus presents a major technical challenge. Here, we demonstrate for the first time that different RMS variants expressed by GAS give rise to genotype-specific and methylome-dependent variation in transformation efficiency. Furthermore, we show that the magnitude of impact of methylation on transformation efficiency elicited by RMS variant TRD_AG_, encoded by all sequenced strains of the dominant and upsurge-associated *emm*1 genotype, is 100-fold greater than for all other TRD tested and is responsible for the poor transformation efficiency associated with this lineage. In dissecting the underlying mechanism, we developed an improved GAS transformation protocol, whereby the restriction barrier is overcome by the addition of the phage anti-restriction protein Ocr. This protocol is highly effective for TRD_AG_ strains including clinical isolates representing all *emm*1 lineages and will expedite critical research interrogating the genetics of *emm*1 GAS, negating the need to work in an RMS-negative background. These findings provide a striking example of the impact of RMS target sequence variation on bacterial transformation and the importance of defining lineage-specific mechanisms of genetic recalcitrance.

**IMPORTANCE:**

Understanding the mechanisms by which bacterial pathogens are able to cause disease is essential to enable the targeted development of novel therapeutics. A key experimental approach to facilitate this research is the generation of bacterial mutants, through either specific gene deletions or sequence manipulation. This process relies on the ability to transform bacteria with exogenous DNA designed to generate the desired sequence changes. Bacteria have naturally developed protective mechanisms to detect and destroy invading DNA, and these systems severely impede the genetic manipulation of many important pathogens, including the lethal human pathogen group A *Streptococcus* (GAS). Many GAS lineages exist, of which *emm*1 is dominant among clinical isolates. Based on new experimental evidence, we identify the mechanism by which transformation is impaired in the *emm*1 lineage and establish an improved and highly efficient transformation protocol to expedite the generation of mutants.

## OBSERVATION

Genetic manipulation of pathogenic bacteria is an essential tool to characterize virulence mechanisms and develop new therapies. However, research into certain pathogens is hampered by their inherent resistance to genetic transformation. One such species is group A *Streptococcus* (GAS), an obligate human pathobiont and the causative agent of a diverse array of infections ranging from pharyngitis and scarlet fever to necrotizing fasciitis (1). Genotype *emm*1 GAS is responsible for an ongoing and unprecedented global surge in infections, the molecular basis for which remains unknown (2 - 5). The development of an improved transformation protocol for *emm*1 GAS is thus essential to expedite critical research into the pathophysiology of this genotype.

The genetic recalcitrance of GAS has been linked to the activity of a chromosomally encoded type 1 restriction modification system (RMS) that is conserved across all genotypes (6 - 8). Type 1 RMS comprise three genes, a DNA restriction endonuclease (*hsdR*), methyl-transferase (*hsdM*), and DNA specificity protein (*hsdS*), which combined form a holoenzyme with methyl-transferase and DNA-cleaving activity (9). The HsdS protein defines the target DNA sequence motif through two distinct 5′ and 3′ target recognition domains (TRDs). Thirteen distinct GAS TRD combinations have been identified, each of which targets a unique DNA motif and is associated with a specific subset of genotypes (6). Interestingly, all *emm*1 strains are associated with a single TRD combination, designated TRD_AG_ (6). While targeted deletion of the RMS and naturally occurring, inactivating mutations have been demonstrated to enhance the transformation efficiency of three GAS genotypes (6 - 8), a direct comparison of the impact of the different methylation patterns conferred by each TRD combination on transformation efficiency has not been performed.

### Methylation-dependent DNA restriction drives the low transformation efficiency of *emm*1 GAS

**Fig 1:**
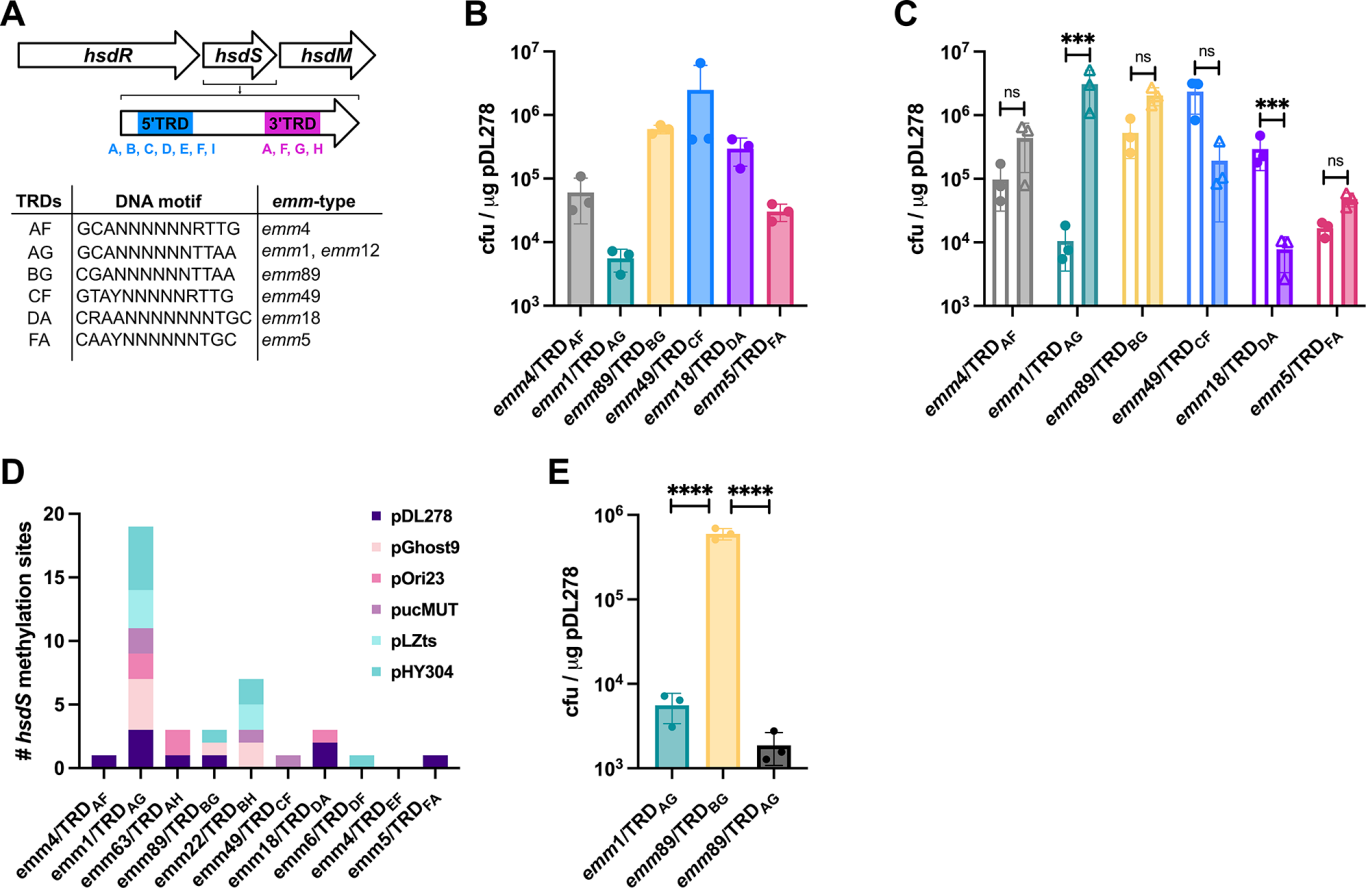
DNA methylation defines the reduced transformation efficiency of *emm*1 GAS. (**A**) Schematic representation of the GAS type 1 RMS and location of TRDs (5′ = blue, 3′ = pink) in the *hsdS* gene (M5005_Spy1622). Each TRD variant identified to date is listed underneath. The table shows six TRD combinations associated with GAS genotypes most frequently subjected to experimental genetic manipulation with details of DNA target motifs (6) and representative associated genotypes used in this study. (**B**) Transformation efficiency of a representative isolate from each of the six TRD combinations highlighted in (**A**) with plasmid pDL278. (**C**) Comparison of the transformation efficiency of a representative isolate from each of the six TRD combinations highlighted in (**A**) with DH5α- (clear bars) or self-methylated (filled bars) plasmid pDL278. Transformation efficiency of *emm*1/TRD_AG_ isolate was enhanced with self-methylated plasmid. Data represent the mean and standard deviation of three independent experiments (multiple unpaired *t*-test analysis on log-transformed data; ***P < 0.001, ns = *P* > 0.05). (**D**) Quantification of DNA target motifs for all GAS TRDs in each of six commonly used plasmids for genetic manipulation of streptococcal pathogens. The target sequence for *emm*1/TRD_AG_ (5′-GCANNNNNNTTAA-3′) was identified more frequently than all other target sequences across all six plasmids. (**E**) Comparison of the transformation efficiency of *emm*1/TRD_AG_ and *emm*89/TRD_BG_ with the “TRD-swap” strain, *emm*89/TRD_AG_ with plasmid pDL278 purified from DH5α. Transformation efficiency of the *emm*89/TRD_BG_ strain was reduced to levels equivalent to *emm*1/TRD_AG_ following the swap of TRD_B_ to TRD_A_. Data represent the mean and standard deviation of three independent experiments (one-way ANOVA test with multiple comparisons performed on log-transformed data; ****P < 0.0001).

In order to determine whether GAS transformation efficiencies differ due to the activity of different TRD variants, transformation efficiencies were quantified for strains representing genotypes most frequently subjected to genetic manipulation for six TRD combinations (Fig. 1A; Table S1). Interestingly, the tested genotypes segregated into two discrete groups with low [10^4^ cfu/µg DNA, *emm*4/TRD_AF_, *emm*1/TRD_AG_ (10), *emm*5/TRD_FA_ (11)] or high [10^6^ cfu/µg DNA, *emm*89/TRD_BG_ (12), *emm*49/TRD_CF_ (13), *emm*18/TRD_DA_ (14)] transformation efficiencies (Fig. 1B). We hypothesized that this difference was a methylation-dependent phenomenon driven by variation in the target sequence for each TRD combination.

All transformations were performed using plasmid DNA purified from the DH5α strain of *Escherichia coli* lineage K12. K12 *E. coli* encodes three methyltransferases (Dam_GATC, Dcm_CCWGG, and EcoKI_AACNNNNNNGTGC/GCACNNNNNNGTT) that target DNA sequences distinct from all known GAS TRDs. Plasmid purified from DH5α will thus be susceptible to cleavage by all GAS TRD variants, where target sites are present. In order to ascertain whether TRD-specific methylation was responsible for the observed differences in transformation efficiency between GAS TRD variants, we tested whether self-methylated plasmid purified from each GAS strain, and thus protected from self-RMS cleavage, impacted DNA uptake (Fig. 1C). Strikingly, *emm*1/TRD_AG_ transformation efficiency was increased 100-fold using self-methylated plasmid. Surprisingly, with the exception of *emm*18/TRD_DA_ for which there was a 10-fold reduction in transformation efficiency, no difference was observed for any other strains tested, including those with an equivalent transformation efficiency to that of *emm*1/TRD_AG_ using DH5α-purified plasmid. While the impaired transformation efficiency of *emm*18/TRD_DA_ was unexpected, we anticipate that this results from deleterious strain-dependent differences in DNA topology (15) or methylation (16). The uniquely improved transformation efficiency observed for *emm*1/TRD_AG_ led us to hypothesize that the activity of the type 1 RMS is higher for *emm*1/TRD_AG_ strains or that more TRD_AG_ recognition motifs are present in the plasmid DNA.

### Unique methylation motif of TRD_AG_ is common in plasmids used for bacterial genetic engineering

The absence of restriction targets in plasmid DNA sequences is an important RMS evasion strategy (17). In order to determine whether the absence of target sequences could explain the unique methylation-associated phenotype for *emm*1/TRD_AG_ GAS, we first compared the frequency of each known GAS TRD recognition motif in plasmid pDL278 (18) used for all experiments, and then expanded our analysis to include five additional commonly used laboratory plasmids (12, 19 - 22) (Table S2). Unexpectedly, the *emm*1/TRD_AG_ recognition sequence was overrepresented across all six plasmids (Fig. 1D) and thus likely contributes to the low transformation efficiency observed for *emm*1/TRD_AG_ GAS.

While overrepresentation of the *emm*1/TRD_AG_ recognition site may completely explain the low transformation efficiency of this lineage, the low efficiency observed for *emm*4/TRD_AF_ and *emm*5/TRD_FA_ strains indicates that other factors also contribute to genetic recalcitrance. We hypothesized that lineage-specific variation in RMS gene sequences or expression levels may also contribute to resistance to transformation, and went on to generate a “TRD-swap” strain in our representative *emm*89/TRD_BG_ isolate to quantitatively assess this possibility. The *emm*1/TRD_AG_ and *emm*89/TRD_BG_ *hsdS* alleles share the same 3′ TRD. Allelic exchange mutagenesis was performed to swap the *emm*89 5′ TRD_B_ with TRD_A_ to generate the TRD_AG_ allele in the *emm*89/TRD_BG_ background, giving rise to strain *emm*89/TRD_AG_. This swap reduced the transformation efficiency of the *emm*89 strain by 100-fold to levels observed for *emm*1/TRD_AG_ (Fig. 1E), a result that strongly implicates overrepresentation of the *emm*1/TRD_AG_ motif as the basis for the poor transformation efficiency of *emm*1/TRD_AG_ strains.

### Inhibition of *emm*1/TRD_AG_ type 1 RMS with phage protein Ocr enhances transformation efficiency

**Fig 2:**
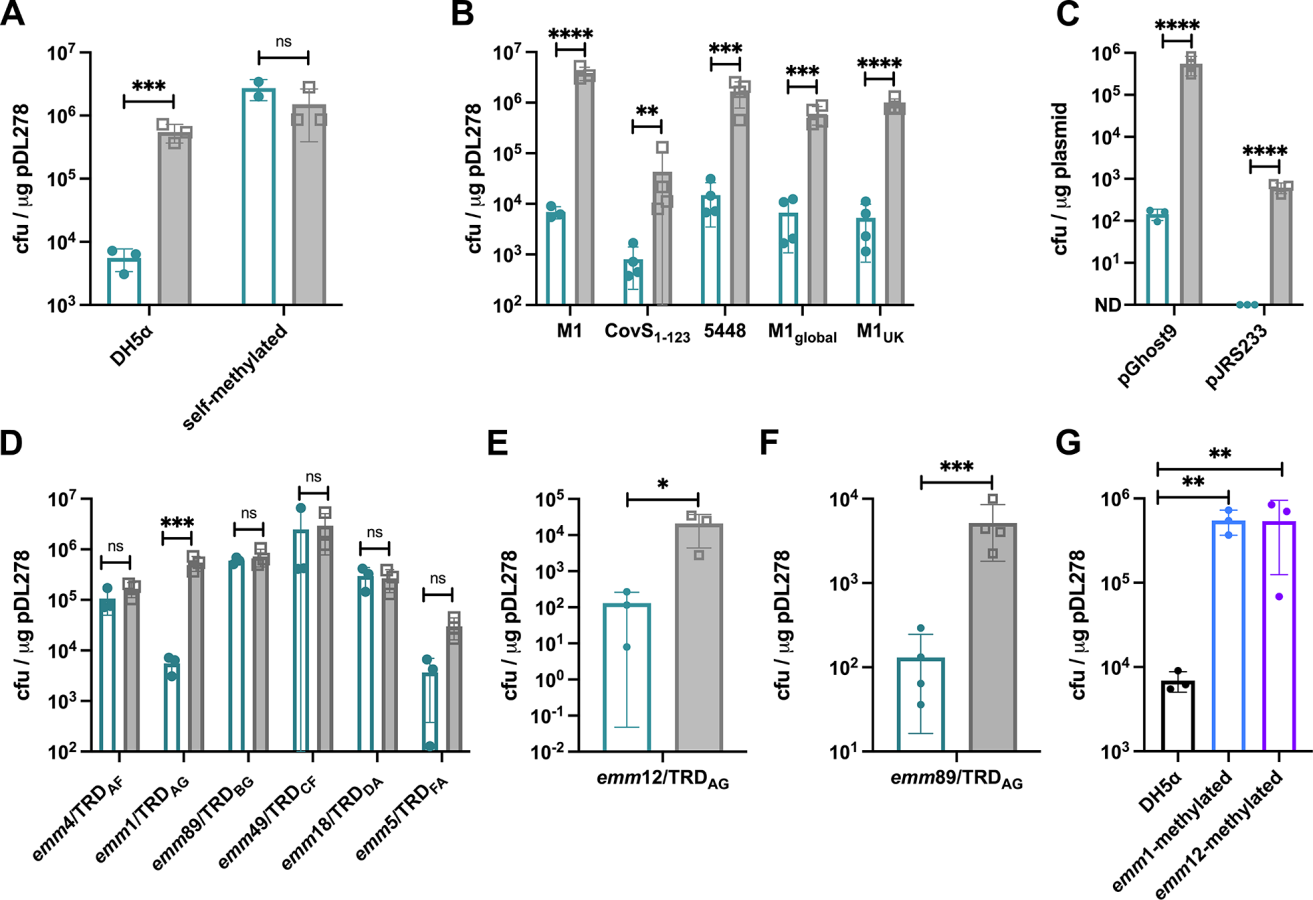
Transformation efficiency of *emm*1 GAS is enhanced 100-fold by inhibition of HsdR with phage protein Ocr. (**A**) Quantification of the transformation efficiency of *emm*1/TRD_AG_ with DH5α- or self-methylated plasmid (pDL278) in the presence/absence of recombinant Ocr protein (clear green bars = −Ocr; filled gray bars = +Ocr). Ocr enhanced the transformation efficiency of *emm*1/TRD_AG_ with DH5α-methylated pDL278 to levels equivalent to self-methylated plasmid, however had no impact on the efficiency of transformation with self-methylated plasmid. Data represent the mean and standard deviation of three independent experiments (multiple unpaired *t*-test analysis on log-transformed data; ***P < 0.001, ns = *P* > 0.05). (**B**) Quantification of the transformation efficiency of 5 *emm*1 isolates representing all major clinically relevant and globally disseminated strains/lineages with DH5α-purified plasmid pDL278 ± recombinant Ocr (clear green bars = −Ocr; filled gray bars = +Ocr). Ocr improved transformation efficiency with plasmid pDL278 100-fold for all strains. Data represent the mean and standard deviation of three independent experiments (multiple unpaired *t*-test analysis on log-transformed data; **P < 0.01, ***P < 0.001, ****P < 0.0001). (**C**) Quantification of the transformation efficiency of strain *emm*1/TRD_AG_ with chromosomal allelic exchange vectors pGhost9 and pJRS233. Plasmids were purified from DH5α and transformed ± recombinant Ocr protein (clear green bars = −Ocr; filled gray bars = +Ocr). Addition of Ocr enhanced transformation efficiency 10^4^- and 10^3^-fold, respectively. Data represent the mean and standard deviation of three independent experiments (multiple unpaired *t*-test analysis on log-transformed data; ****P < 0.0001). (**D**) Quantification of the transformation efficiency of a representative isolate from each of the six TRD combinations highlighted in Fig. 1A with plasmid pDL278 purified from DH5α ± recombinant Ocr protein (clear green bars = −Ocr; filled gray bars = +Ocr). Addition of Ocr had no effect on the transformation efficiency of any strain other than *emm*1/TRD_AG_. Data represent the mean and standard deviation of three independent experiments (multiple unpaired *t*-test analysis on log-transformed data; ***P < 0.001, ns  = *P* > 0.05). (**E**) Quantification of the transformation efficiency of *emm*12/TRD_AG_ with plasmid pDL278 purified from DH5α ± recombinant Ocr (clear green bars = −Ocr; filled gray bars = +Ocr). Ocr enhanced transformation efficiency 100-fold. Data represent the mean and standard deviation of three independent experiments (unpaired *t*-test analysis on log-transformed data; *P < 0.05). (**F**) Quantification of the transformation efficiency of *emm*89/TRD_AG_ with plasmid pDL278 purified from DH5α ± recombinant Ocr (clear green bars = −Ocr; filled gray bars = +Ocr). Ocr enhanced transformation efficiency 100-fold. Data represent the mean and standard deviation of three independent experiments (multiple unpaired *t*-test analysis on log-transformed data; **P < 0.01). (**G**) Quantification of the transformation efficiency of *emm*1/TRD_AG_ with plasmid pDL278 purified from DH5α, *emm*1/TRD_AG_, and *emm*12/TRD_AG_. Transformation of *emm*1/TRD_AG_ with plasmid purified from either GAS strain expressing TRD_AG_ was enhanced by 100-fold. Data represent the mean and standard deviation of three independent experiments (one-way ANOVA test with multiple comparisons performed on log-transformed data; ****P < 0.0001).

Having shown that the reduced transformation efficiency associated with *emm*1/TRD_AG_ could be explained by overrepresentation of TRD_AG_ target motifs in commonly used plasmids, we went on to determine whether inhibition of the type 1 RMS with the phage anti-restriction protein Ocr (23) could improve the transformation efficiency of this genotype without the need to generate an isogenic RMS deletion mutant. The addition of Ocr protein (50 ng/µL) to the electroporation reaction enhanced the efficiency 100-fold following transformation with DH5α-purified plasmid, equivalent to the highest efficiencies observed for *emm*89/TRD_BG_ strains but had no effect on transformation with self-methylated plasmid (Fig. 2A).

In order to confirm that this was not a strain-specific phenomenon, we expanded the isolates tested to include *emm*1 strains representing the most common genetic variants; a naturally occurring CovS_1–123_ mutant (10), M1T1 strain 5448 (USA origin) (24), and strains representing the two dominant lineages circulating currently and responsible for the current upsurge in GAS infections globally, M1_global_ (5) and M1_UK_ (5). Transformation of all *emm*1 strains, each encoding TRD_AG_, was improved by the same magnitude following the incorporation of Ocr into the transformation reaction (Fig. 2B). This result demonstrates that the transformation efficiency of multiple *emm*1 clinical isolates from diverse lineages is greatly enhanced using this protocol.

In order to confirm that this effect was not specific to the pDL278 plasmid, we performed similar experiments with plasmids pGhost9 (19) and pJRS233 (25), frequently used as suicide vectors. The addition of Ocr protein enhanced the transformation efficiency of *emm*1/TRD_AG_ with both plasmids by an even greater magnitude than that observed for pDL278 (Fig. 2C).

### Ocr enhancement of GAS transformation is restricted to strains expressing TRD_AG_

We went on to ascertain whether the addition of Ocr was sufficient to enhance the transformation efficiency of strains representing other TRD combinations. As observed for transformation with self-methylated plasmid (Fig. 1C), Ocr did not significantly enhance the transformation efficiency of any non-*emm*1/TRD_AG_ genotypes (Fig. 2D), likely due to reduced representation of unique genotype-specific RMS target sites in plasmid pDL278 (Fig. 1D). Importantly, similar to *emm*1 GAS, the efficiency of DNA uptake by an *emm*12/TRD_AG_ strain (Fig. 2E) and the *emm*89/TRD_AG_ (Fig. 2F) was enhanced two log-fold following the addition of Ocr protein, indicating that this phenotype is methylation-dependent. This conclusion is further supported by the observation that transformation of *emm*1/TRD_AG_ GAS was similarly enhanced using plasmid purified from and methylated by either *emm*1/TRD_AG_ or *emm*12/TRD_AG_ GAS (Fig. 2G).

Together, these data demonstrate that the type 1 RMS prevents efficient transformation of *emm*1 GAS due to overrepresentation of the target methylation site in plasmids, and that this restriction barrier can be overcome by the addition of the phage protein Ocr. We go on to establish an experimental protocol for enhanced transformation of TRD_AG_ GAS that is effective for diverse clinical *emm*1 strains.

## Data Availability

All relevant data are within the manuscript and Supplemental Materials files.

## SUPPLEMENTAL MATERIAL

Supplemental text mbio.00798-23-s0001.pdf Supplemental methods.

Tables S1 to S3 mbio.00798-23-s0002.pdf Strains, plasmids, and primers used in this study.

ASM does not own the copyrights to Supplemental Material that may be linked to, or accessed through, an article. The authors have granted ASM a non-exclusive, world-wide license to publish the Supplemental Material files. Please contact the corresponding author directly for reuse.
