# Supplementary material for: Methylome-dependent transformation of emm1 group A streptococci
Source: mBio. 2023 Jul 10;14(4):e00798-23. doi: 10.1128/mbio.00798-23 (PMC10470502; doi:10.1128/mbio.00798-23)
Supplement: Supplemental text — Supplemental methods. [file mbio.00798-23-s0001.pdf]

Supplemental Methods

Bacterial strains and growth conditions

GAS clinical strains (Table S1) were cultured on trypticase soy agar supplemented with 5% defibrinated sheep blood (R&D) or Todd-Hewitt (TH) (Oxoid) agar or in TH broth at 37°C. *E. coli* strain DH5α (New England Biolabs) was used for storage and passaging of all plasmids and cloning. *E. coli* was cultured on LB agar (Formedium) or broth (Sigma) at 37°C with shaking at 180 rpm. Growth media were supplemented with antibiotics where appropriate at the following concentrations: for *E. coli*, spectinomycin (Sigma) at 50 µg/ml, erythromycin (Sigma) at 250 µg/ml; for GAS, spectinomycin at 50 µg/ml and erythromycin at 1 µg/ml.

Sanger sequencing of the *hsdS* gene

Genomic DNA was extracted from GAS cultures grown to late logarithmic growth phase (OD_{600} 0.7–0.9) as described previously(14). PCR was carried out with primers *hsdS* F and *hsdS* R (Table S3) with Phusion Flash High-Fidelity PCR Master Mix (Thermo) using a MyCycler (Bio-Rad) thermal cycler. Sanger sequencing was performed in order to determine the sequence of the *hsd*S gene.

Transformation protocol

<u>Preparation of competent cells</u>: GAS were cultured to OD_{600} 0.2 in 40 ml THB and pellets washed 5 times in 1 ml ice-cold 0.5 M sucrose (Sigma) (16000 xg, 1 minute, 4°C). Pellets were re-suspended in a final volume of 100 µl ice-cold 0.5 M sucrose and 50 µl aliquots were used immediately for electrotoporation(7).

<u>Electroporation</u>: 5 µl plasmid DNA (at 100 ng/µl) was mixed with 50 µl competent cells and stored on ice. DNA was transformed by electroporation (MicroPulser Electroporator, Biorad) with the following settings: 200 Ω, 1.7 kV, 50 µF, 0.1 cm cuvette (Thermo). Cells were recovered in 1 ml THB, cultured for 1 hour at 37°C for pDL278 or 2 hours at 30 for pJRS233 and pGhost9, and plated on selective media(7).

Where relevant 1 µl (2.5 µg) Ocr (TypeOne^{TM}, Lucigen) was added to electroporation reactions prior to pulsing.

Purification of GAS self-methylated plasmid

Plasmid DNA was purified from successfully transformed GAS strains using a modified QIAprep Spin Miniprep (Qiagen) purification protocol. Briefly, GAS were cultured overnight in 50 ml THB and pellets resuspended in 1 ml QIAprep buffer P1 supplemented with mutanolysin (100 units/ml, Sigma) and lysozyme (1 mg/ml), and incubated for 30 minutes at 37°C. Lysates were divided into 4x 250 µl aliquots and mixed with buffers P2 and N3 as per manufacturers guidelines. Following a 10-minute centrifugation step, supernatants were concentrated 2-fold and then purified twice over sequential QIAprep columns. Plasmid DNA was eluted in 50 µl nuclease-free water/column.

Generation of TRD_{AG}/TRD_{BG} swap strain

Using the temperature-sensitive *E. coli*-GAS shuttle vector pJRS233(25), plasmid pJRS_hsdS_M89/M1_swap was generated to facilitate creation of an isogenic *emm*89/TRD_{BG} strain expressing the TRD_{AG} *hsdS* allele, where the 5' TRD_A was swapped with TRD_B. Primers hsdS_swap_F1 and _R1 (Table S3) were used to amplify the 5' TRD_A sequence from genomic DNA purified from *emm*1 GAS. A region of DNA downstream of the TRD was necessary to facilitate homologous recombination within the *emm*89 genome. Due to a single SNP in the 3' homologous region, this second amplicon was amplified from *emm*89 genomic DNA using primers hsdS_swap_F2 and _R2 (Table S3) and spliced to 5' TRD_A by overlap extension (SOEing) PCR, using overlapping the amplicons as target DNA. This was performed using primers hsdS_swap_F1 and _R2 (Table S3), incorporating the restriction sites BamHI and SalI into the resulting PCR product to facilitate cloning into the vector pJRS233. The resulting shuttle vector pJRS_hsdS_M89/M1_swap was transformed into *emm*89/TRD_{BG} GAS by electroporation, and 5'TRD_A was exchanged with the chromosomal copy of 5'TRD_B by allelic-exchange

mutagenesis. Sanger sequencing was performed to confirm introduction of TRD_{AG} into the chromosomal TRD_{BG} gene.

Statistics

All statistical analyses were performed with GraphPad Prism 9. Data were log-transformed, and comparisons of two datasets were carried out using an unpaired t-test or "multiple unpaired t-tests" where more than one comparison was performed for datasets on a single graph. Comparisons of more than two datasets were carried out using a one-way ANOVA. A p-value of <0.05 was considered significant.
