# Supplementary material for: Methylome-dependent transformation of emm1 group A streptococci
Source: mBio. 2023 Jul 10;14(4):e00798-23. doi: 10.1128/mbio.00798-23 (PMC10470502; doi:10.1128/mbio.00798-23)
Supplement: Tables S1 to S3 — Strains, plasmids, and primers used in this study. [file mbio.00798-23-s0002.pdf]

Supplemental Tables

Table S1. Strains used in this study

Strain	Description	Reference/source
*emm*1/TRD$_{AG}$	Identical to H584	(10)
*emm*1_CovS$_{1\text{-}123}$	Identical to H598	(10)
*emm*1_5448	Identical to 5448	(24)
*emm*1$_{global}$	Identical to H1489	(5)
*emm*1$_{UK}$	Identical to H1490	(5)
*emm*4/TRD$_{AF}$		Kind Gift, CE Turner
*emm*5/TRD$_{FA}$	Identical to Manfredo	(11)
*emm*12/TRD$_{AG}$	Identical to H873	Kind gift, S Sriskandan
*emm*18/TRD$_{DA}$	Identical to H566	(14)
*emm*49/TRD$_{CF}$	Identical to NZ131	(13)
*emm*89/TRD$_{BG}$	Identical to H293	(12)
*emm*89/TRD$_{AG}$	5'TRD$_{BG}$ to 5'TRD$_{AG}$	This study

Table S2. Plasmids used in this study

Strain	Reference/source
pDL278	(18)
pGhost9	(19)
pOri23	(20)
pUCMUT	(12)
pLZts	(21)
pHY304	(22)

Table S3. Primers used in this study

Primer name	Sequence 5'-3'*
hsdS F	GGTGATTCTGCCATGAATAAAC
hsdS R	GGCCGAATGCCATAGTGCTTG
hsdS_swap_F1	CG**GGATCC**ATCCAAAGGGCCATCAAAC
hsdS_swap_R1	GGAAAAGTTCGCCAATGGC
hsdS_swap_F2	GCCATTGGCGAACTTTTCC
hsdS_swap_R2	CGCC**GTCGAC**GAGGAAATATTAATATCTGG

*Bold with underlining indicates restriction endonuclease sites.
